# Branched chain amino acids alter fatty acid profile in colostrum of sows fed a high fat diet

**DOI:** 10.1186/s40104-019-0423-9

**Published:** 2020-02-17

**Authors:** Chang Ma, Yajng Liu, Shaoshuai Liu, Crystal L. Lévesque, Fengqi Zhao, Jindong Yin, Bing Dong

**Affiliations:** 1 State Key Laboratory of Animal Nutrition, College of Animal Science and Technology, China Agricultural University, Beijing, 100193 China; 2 Department of Animal Science, College of Agriculture and Biological Sciences, South Dakota State University, Brookings, SD 57007 USA; 3 Department of Animal and Veterinary Sciences, University of Vermont, Burlington, VT 05405 USA

**Keywords:** Dietary lipids, Fatty acid composition, Milk, Odd chain fatty acid, Pig, Poly unsaturated fatty acid, Sow

## Abstract

**Background:**

Branched chain amino acids (BCAAs) are important substrates for milk protein synthesis in the mammary gland, and are tightly related to lipid metabolism. No study has been performed examining the role of BCAAs with high fat diets on milk fat synthesis. This study was designed to investigate the effect of dietary BCAAs on growth performance of piglets, progeny body weight, and milk fat composition in sows fed a high fat diet. Four diets (CON = control diet; HF = high fat diet with 8% soybean oil; HF-MB=HF plus 0.39% BCAAs; HF-HB=HF plus 0.78% BCAAs) were fed to sows from late gestation to weaning.

**Results:**

Compared to HF, BCAAs (HF-MB and HF-HB) increased the litter weight (*P* < 0.05) and overall litter weight gain (*P* < 0.05) at weaning and increased colostrum fat content by 27.3–35.8% (*P* < 0.01). Fatty acid profiles between the two doses of BCAAs were similar. Compared with HF, HF-MB tended to decrease the percentage of C18:3n3 (*P* = 0.063) and increased the percentage of C18:1n9c (*P* = 0.03). In addition, BCAAs in HF-MB increased the concentration of total fatty acid by 22.1% in colostrum (*P* = 0.03) but decreased that in serum at parturition by 53.2% (*P* = 0.027). The fatty acids in colostrum that increased with BCAAs were C15:0, C17:0, C20:3n6, C20:4n6, C20:5n3 and C22:6n3 (*P* = 0.00~0.04). Colostrum fatty acids of C20:0, C21:0, C22:0, C16:1, C20:1, C18:1n9c also tended to be increased (0.05 < *P* < 0.1) with BCAAs. The change in sow serum fatty acid profile due to BCAAs was different from that in colostrum.

**Conclusions:**

BCAAs in high fat diet of sows altered the fatty acid composition in colostrum and enhanced litter growth. Our study indicated that BCAAs supplementation can enhance mammary fatty acid uptake and mammary fat synthesis and that supplemental BCAAs and fat in late gestation and lactation diets for sows can improve reproductive performance.

## Background

In modern swine husbandry, sow performance is largely dependent on litter size and survival rates. Insufficient energy supply in gestation and lactation is a major limitation for high sow performance. Dietary addition of fat is often used to increase sow energy intake during late gestation and lactation. It has been shown that milk fat, piglet survival rate and weaning weight are increased when supplemental fat is raised to 8% [1]. Several studies have demonstrated supplemental fat in maternal diets improved the survival rate of piglets, in particular small piglets compared to bigger littermates (reviewed by [2]).

BCAAs, composed of leucine, isoleucine and valine, are neutral essential amino acids and represent 35% of the essential amino acids in food. BCAAs are also the nitrogenous precursors for the synthesis of glutamine, alanine and aspartate [3]. In the mammary gland, mammary epithelial cells can catabolize BCAAs by BCAA transaminase to branched-chain α-keto acids (BCKA). During lactation, the mammary gland contains both active branched-chain aminotransferase and BCKA dehydrogenase complex (BCKD) [4]. BCKD can further decarboxylate BCKA into a series of metabolites that enter the TCA cycle to produce energy [5]. Notably, BCAAs are potentially important to mammary gland lipid synthesis owing to their tight relation with lipid metabolism. The kinase BCKD kinase (BDK) and a phosphatase are two regulators of active/inactive form of BCKD. Overexpression of BDK in liver activated *de novo* lipogenesis [6]. Thus BDK as a node integrates BCAAs metabolism and lipogenesis. The catabolic intermediate of valine, 3-hydroxy-isobutyrate, is a paracrine regulator of fatty acid flux [7]. Feeding high fat diets with the inclusion of BCAA to rodents reduces obesity and hepatocyte fat deposition [8]. In finishing pigs, isoleucine supplementation increase lipogenesis in intramuscular fat [9]. Furthermore, BCAAs play functional roles in the female animal during lactation. Valine is the third limiting amino acids for lactating sows after lysine and threonine [10]. An optimized valine to lysine ratio in sow diets minimized sow backfat loss during lactation and maximized piglet growth [11]. During late gestation, supplementing isoleucine and valine in sow diets increased litter weights [12]. Furthermore, feeding dairy cows with the metabolites of leucine, isovaleric acid [13] and α-ketoisocaproic acid [14], increased milk fat. The leucine metabolite, β-hydroxy-β-methyl butyrate which is approximately 5% of the metabolites from leucine oxidation, improved sow backfat at farrowing and increased colostrum milk fat [15].

Because BCAAs affect sow performance and are associated with lipid metabolism, we hypothesize that BCAAs supplementation in a high fat diet of sows can effectively improve milk fat production and reproductive performance of sows. In this study, we investigated the effects of supplementation with two doses of BCAAs on backfat, progeny body weights, and colostrum in sows.

## Materials and methods

All animal procedures used in this study were approved by the Institutional Animal Care and Use Committee of China Agricultural University (Beijing, China). The experiment was performed at the National Feed Engineering Technology Research Center of Ministry of Agriculture Feed Industry Center Animal Testing Base (Hebei, China).

### Experimental design and diets

Forty-eight multiparous sows (Large White × Landrace, body weight: 252.57 ± 23.14 kg, and parity: 3.34 ± 1.37) were used in this study. This study was conducted over 3 winter months in North China. The temperature of pig houses was kept at 15-25 °C with electronic heaters. On d 107 of gestation, sows were weighed and backfat thickness was measured. The sows were assigned to four blocks based on their body weight, backfat thickness and parity. Each block was randomly allotted to one of four dietary treatment groups: Control group (CON, *n* = 8), High fat group (HF, *n* = 16), High fat with medium dose of BCAAs (HF-MB, *n* = 16), and High fat with high dose of BCAAs (HF-HB, *n* = 8). The primary purpose of the study was the comparison of HF and HF-MB based on a study conducted by our group using pregnant rats suggesting a difference in mammary gland fatty acid concentration with medium dose of BCAAs addition to high fat diets. The CON and HF-HB treatments were added for enhanced treatment comparison; the number of sows/treatment for CON and HF-HB were based on the confines of sow availability at the pig farm. Full details of the rat study will be submitted elsewhere. Additional file 1: Figure S1 provides summary of relevant response. The dietary treatment was conducted from d 107 of gestation to d 24 of lactation. On d 107 of gestation, sows were moved to individual farrowing crates (2.0 m × 3.0 m) in environmentally controlled pig houses until weaning. Lights were on from 06:00 to16:00 h. Sows were weighed on d 107 of gestation, within 48 h of farrowing, and on d 24 of lactation in order to calculate body weight loss. Back fat thickness (P2, 6 cm from the midline at the head of the last rib) was measured on d 107 of gestation, at farrowing and on d 24 of lactation with an ultrasonic device (Piglog105; SFK Technology A/S, Herlev, Denmark). Within 24 h of farrowing, litter size was standardized to 10–11 piglets per sow by cross-fostering within the same treatment. Body weights of piglets were recorded at birth and weaning to calculate average daily weight gain (ADG). On d 1 of birth, piglets were processed according to standard husbandry practices that included 200 mg of iron (iron dextran solution, intramuscular injection), ear notching and clipping needle teeth and tails. Male piglets were castrated on d 7.

Sows were provided experimental diets at 2.0 kg/d experimental diets from d 107 of gestation to parturition. After farrowing, daily feed allocation was progressively increased to d 5 of lactation with ad libitum access to feed from d 6 until weaning. Experimental diets were provided three times daily at 06:00, 11:00 and 16:00 h. Total feed intake was recorded to calculate average daily feed intake (ADFI).

The compositions and nutrient levels of four experimental diets are listed in Table 1. The fatty acid compositions of experimental diets are shown in Table 2. The CON diet was based on corn-soybean meal without soybean oil. The HF diet contained 8% soybean oil according to the recommended supplementation levels [1]. The HF-MB and HF-HB diets were the HF diet supplemented with a medium dose (0.11% leucine, 0.06% isoleucine and 0.22% valine or a high dose (0.22% leucine, 0.12% isoleucine and 0.44% valine) of BCAAs, respectively. The amounts of BCAAs supplemented in HF-HB were double than those in HF-MB diet. The dose of supplemental BCAAs in HF-MB was set according to a previous report that total BCAAs of 2.85% [12], and optimal ratio of valine to lysine was 94% [11]. The ratios of leucine, isoleucine and valine were kept approximately 2:1:1.5 as per NRC (2012) recommendations for lactating multiparous sows. The levels of BCAAs in both HF-MB and HF-HB diets met or exceeded the amino acid requirements for multiparous sows during lactation. All four diets were isonitrogenous and had similar levels of other amino acids. The protein and energy contents in all diets met the requirement for multiparous sows by NRC (2012). The analyzed content of leucine, isoleucine and valine in HF-MB diet were 1.38, 0.62% and 0.85% respectively, while they were 1.49, 0.68% and 1.07% respectively in HF-HB diet. The formulated digestible energy of CON was 13.81 MJ/kg, lower than HF, HF-MB and HF-HB which were 15.59, 15.56 and 15.51 MJ/kg, respectively. All diets were prepared fresh weekly. Feed samples of every batch were retained and samples pooled within dietary treatment. They were stored at − 20 °C until analysis.

**Table 1:** Ingredient composition and nutrient levels of experimental diets (as-fed basis, %)

Items^a^	CON	HF	HF-MB	HF-HB
Corn	69.77	61.77	61.52	61.26
Soybean meal	10.00	10.00	10.00	10.00
Wheat bran	7.00	7.00	7.00	7.00
Peanut meal	10.40	10.40	10.40	10.40
Soybean oil	0.00	8.00	8.00	8.00
Dicalcium phosphate	0.80	0.80	0.80	0.80
Limestone	1.00	1.00	1.00	1.00
Sodium chloride	0.30	0.30	0.30	0.30
*L*-Lys·HCl (78.8%)	0.23	0.23	0.23	0.23
*L*- Leucine	0.00	0.00	0.22	0.44
*L*-Isoleucine	0.00	0.00	0.06	0.12
*L*-valine	0.00	0.00	0.09	0.17
Premix^b^	0.50	0.50	0.50	0.50
Nutrient levels^c^				
Digestible energy, MJ/kg	13.81	15.59	15.56	15.51
Crude protein	16.74	16.01	16.16	15.38
Calcium	0.69	0.71	0.70	0.69
Total phosphorus	0.61	0.60	0.61	0.63
Lysine	0.92	0.90	0.90	0.90
Methionine + cysteine	0.56	0.53	0.53	0.53
Threonine	0.62	0.60	0.60	0.60
Tryptophan	0.18	0.17	0.18	0.17
Leucine	1.35	1.27	1.38	1.49
Isoleucine	0.58	0.56	0.62	0.68
Valine	0.55	0.54	0.85	1.07

^a^Experimental diets were a corn-soybean meal based diet (CON), CON + 8% soybean oil (HF), HF + medium dose of branch chain amino acids (BCAAs, 0.11% leucine, 0.06% isoleucine and 0.22% valine; HF-MB), and HF + high dose of BCAAs (0.22% leucine, 0.12% isoleucine and 0.44% valine; HF-HB). ^b^The premix contained (per kg of complete diet): vitamin A, 12000 IU; vitamin D_3_, 2000 IU; vitamin E, 24 IU; vitamin K_3_, 2.0 mg; thiamine, 2.0 mg; riboflavin, 6.0 mg; pyridoxine, 4 mg; vitamin B_12_, 24 μg; niacin, 30 mg; pantothenic acid, 20 mg; folic acid, 3.6 mg; biotin, 0.4 mg; choline chloride, 0.4 mg; iron, 96 mg; copper, 8.0 mg; zinc, 120 mg; manganese, 40 mg; iodine, 0.56 mg; and selenium, 0.4 mg. ^c^All nutrient levels, except digestible energy, were measured

**Table 2:** Fatty acid compositions of experimental diets (g/100 g)

Items^a^	CON	HF	HF-MB	HF-HB
C10:0	0.01	0.02	0.01	0.02
C12:0	0.10	0.10	0.15	0.13
C14:0	0.04	0.15	0.15	0.13
C15:0	0.02	0.09	0.03	0.04
C16:0	6.12	15.61	14.32	13.78
C16:1	0.05	0.13	0.13	0.11
C17:0	0.03	0.19	0.12	0.11
C18:0	0.89	4.63	4.17	4.08
C18:1n9c	10.25	29.63	27.57	25.70
C18:2n6c	20.67	64.88	59.39	58.61
C18:3n3	0.96	6.90	6.39	6.92
C20:0	0.31	0.66	0.65	0.61
C20:1	0.10	0.30	0.30	0.28
C21:0	0.02	0.09	0.07	0.09
C22:0	0.11	0.56	0.50	0.44
C22:1n9	0.02	0.03	0.04	0.05
C20:3n6	0.00	0.02	0.02	0.02
C24:0	0.17	0.35	0.38	0.31
SFA^b^	7.82	22.45	20.55	19.75
MUFA^c^	10.42	30.10	28.12	26.14
PUFA^d^	21.63	71.80	65.81	65.55
Total fatty acids	39.87	124.36	114.48	111.44

^a^Experimental diets were a corn-soybean meal based diet (CON), CON + 8% soybean oil (HF), HF + medium dose of branch chain amino acids (BCAAs, 0.11% leucine, 0.06% isoleucine and 0.22% valine; HF-MB), and HF + high dose of BCAAs (0.22% leucine, 0.12% isoleucine and 0.44% valine; HF-HB). Control diet contained no soybean oil (CON). ^b^*SFA* Saturated fatty acids. ^c^*MUFA* Monounsaturated fatty acids. ^d^*PUFA* Polyunsaturated fatty acids

### Milk and blood sample collections

Colostrum was collected from sows within 2 h of the birth of the first piglet. Milk samples were collected from all functional glands by injection of 1 mg oxytocin into the ear vein in the morning of d 12 and d 18 of lactation before feeding. Sow blood samples were collected by precaval vein puncture on the day of parturition and on d 12 of lactation before the first AM feeding. Blood samples were kept at room temperature to clot followed by centrifugation at 3500 × *g* (Biofuge22R; Heraeus, Hanau, Germany) for 15 min for serum preparation. Colostrum, milk and sera were stored at − 80 °C until analysis.

### Chemical analyses

The analysis of the crude protein (CP), calcium, phosphorus was conducted according to the Association of Official Analytical Chemists Method 990.03, 968.08 and 946.06 (AOAC 2006). All amino acids, except methionine and tryptophan, were analyzed using the methods of AOAC Method 999.13 (AOAC 2003). Methionine and tryptophan were determined using the method of AOAC method 994.12. Milk immunoglobulins were analyzed with commercial kits following manufacturer’s instructions (Sanwei Biological Engineering Co., Ltd., Shandong, China). FA analysis was carried out using the reported method of gas chromatography with modification [16]. Briefly, milk samples were extracted using chloroform: methanol (2:1 v/v) and the extracts evaporated before trans-esterified using 1% H_2_SO_4_ in methanol for 2 h at 70 °C. The resulting methyl esters were extracted and FA methyl esters (FAME) were then separated and quantified using a Shidmadzu 2010 gas chromatograph equipped with a 50-mm capillary column (0.32-mm internal diameter) coated with BPX-70 (0.25-μm film thickness; SGE Pty Ltd., Ringwood, VIC, Australia). Each sample (1 μL) was then injected in to the column using an automatic injector (Shimadzu AOC 20i, Shimadzu Corporation, Kyoto, Japan) at a split ratio of 20:1. Identification of FA peaks was made by comparing their retention times to that of known FAME standards (Sigma-Aldrich, St Louis, MO, USA). C11:0 was set as the internal control by adding to the initial milk sample prior to extraction. Individual FA peaks were quantified as absolute values (mg/mL) and the percentage of the total area under the FA peaks and reported as a percentage of total FAs.

### Statistical analysis

GLM procedure of SAS (SAS Institute Inc., Cary, NC, USA) followed by least significant difference (LSD) method was used to analyze statistical differences among groups. An individual sow or a litter of piglet was used as an experimental unit. Planned orthogonal contrasts were used to evaluate the overall effect of high fat ([CON] vs. [HF, HF-MB, HF-HB]), BCAAs effect ([HF] vs. [HF-MB, HF-HB]) or BCAAs dose effect ([HF-MB] vs. [HF-HB]). All data were checked for normal distribution and homogeneity of variance using the Shapiro-Wilk normality test and Bartlett test, respectively. All values are reported as least squares means. Treatment effects were considered significant if *P* less than 0.05, whereas *P* between 0.05 and 0.10 was considered a trend.

## Results

### Effects of BCAAs on sow and litter performance

Compared to CON diet, the high fat diets (HF, HF-MB and HF-HB) lowered total sow feed intake and average daily feed intake (*P* < 0.05) (Table 3). At weaning, sows provided high fat diets were heavier (*P* = 0.03) than CON, consistent with lesser (*P* < 0.05) BW and backfat loss during lactation. Compared to CON, high fat diets (HF, HF-MB and HF-HB) significantly increased (*P* < 0.05) number of live piglets/litter, live litter weight, and individual piglet weight at birth. Similarly, high fat diets increased litter size at weaning (*P* = 0.01), piglet survival rate (*P* < 0.01), individual pig and litter weight, and overall litter weight gain (*P* < 0.01) at weaning. With respect to BCAAs supplementation to high fat diet, total and individual BCAAs intake was greater (*P* < 0.02) with BCAAs supplementation and with high dose (Table 3). No effect of BCAAs supplementation or dose was detected for sow lactation feed intake, BW or BW loss. Compared to HF, backfat loss was reduced (*P* = 0.02) with BCAAs supplementation. Compared to HF, BCAAs supplementation had no effect on litter and piglet characteristics at birth or piglet survival rate. However, litter and individual piglet weight at weaning and overall litter weight gain increased (*P* < 0.05) and individual pig daily gain tended to increase (*P* = 0.07) with BCAAs supplementation. There was no effect of BCAAs dose on sow or litter performance. (Table 3).

**Table 3:** Effects of branched chain amino acid supplementation on performance of lactating sows fed a high-fat diet^1^

Items	CON	HF	HF-MB	HF-HB	Pooled SEM	*P*-value		
						HF effect^3^	BCAAs effect^4^	BCAAs dose effect^5^
Sows								
No. of sows	8	16	16	8				
Days of experiment	30.4	31.2	31.0	31.6	0.59	0.12	0.24	0.61
Total feed intake, kg (During lactation)	161.60^a^	130.40^b^	126.01^b^	128.05^b^	8.64	0.02	0.30	0.48
ADFI, kg (During lactation)	6.91^a^	5.39^b^	5.25^b^	5.21^b^	0.07	0.01	0.17	0.36
Total BCAA intake, g/d								
Leucine intake	78.03	58.80	62.38	67.05	2.01	0.15	0.01	0.001
Isoleucine intake	33.52	25.93	28.02	30.60	1.65	0.23	0.003	0.001
Valine intake	31.79	25.00	37.97	48.36	1.74	0.38	0.001	0.000
Sow BW change, kg								
BW at farrowing	261.57	264.34	263.69	261.33	6.44	0.74	0.22	0.28
BW at weaning	228.14^a^	238.70^b^	238.77^b^	230.50^ab^	8.94	0.03	0.34	0.42
BW loss	33.43^a^	25.64^b^	24.91^b^	30.83^a^	5.43	0.02	0.67	0.59
Sow backfat change, mm								
Backfat at farrowing	17.27	16.36	15.62	15.80	0.97	0.19	0.17	0.52
Backfat at weaning	15.82	14.86	14.62	14.80	1.16	0.18	0.41	0.44
Backfat change	1.55^a^	1.51^a^	1.08^b^	1.00^b^	0.84	0.03	0.02	0.63
Piglets								
At birth								
Total piglet No. at birth	10.20	10.58	10.61	10.45	0.53	0.16	0.67	0.49
Live piglet No. at birth	10.00^a^	10.52^b^	10.48^b^	10.39^b^	0.41	0.02	0.64	0.37
Litter wt of live piglets, kg	16.18^a^	18.01^b^	18.90^b^	17.72^b^	1.01	0.01	0.26	0.34
Individual BW of live piglet, kg	1.62^a^	1.72^b^	1.80^b^	1.71^b^	0.08	0.03	0.57	0.61
At weaning								
Piglet No. after cross-fostering per litter^2^	10.40	10.40	10.41	10.25	0.09	0.85	0.59	0.63
Weaned piglet No. per litter	9.71^a^	10.28^b^	10.15^b^	10.07^b^	0.18	0.01	0.25	0.28
Weaning survival rate,%	93.45^a^	98.93^b^	97.59^b^	98.30^b^	1.62	0.003	0.43	0.56
Initial body wt per litter, kg	17.58	17.01	18.70	17.90	1.01	0.41	0.55	0.67
Weaned BW per litter, kg	70.71^a^	74.72^b^	80.89^b^	78.72^b^	2.09	0.001	0.03	0.36
Overall litter wt gain, kg	53.05^a^	57.87^ab^	62.20^b^	61.04^b^	2.01	0.002	0.02	0.71
Initial piglet weight, kg	1.68	1.65	1.71	1.75	0.08	0.72	0.59	0.66
Weaned piglet wt, kg	7.35^a^	7.48^a^	7.99^b^	7.88^b^	0.36	0.03	0.04	0.28
ADG, kg	0.241^a^	0.255^b^	0.275^b^	0.262^b^	0.10	0.008	0.07	0.37

^1^Experimental diets were a corn-soybean meal based diet (CON), CON + 8% soybean oil (HF), HF + medium dose of branch chain amino acids (BCAAs, 0.11% leucine, 0.06% isoleucine and 0.22% valine; HF-MB), and HF + high dose of BCAAs (0.22% leucine, 0.12% isoleucine and 0.44% valine; HF-HB). Control diet contained no soybean oil (CON). In the same row, values with different small letter superscripts mean significant difference (*P* < 0.05) when comparing [CON] vs. [HF, HF-MB, HF-HB]. A trend is declared when *P* is no more than 0.10 (0.05 ≤ *P* ≤ 0.1). ^2^Litter size cross-fostering was conducted within treatment. ^3^Orthogonal contrast statement: [CON] vs. [HF, HF-MB, HF-HB]. ^4^Orthogonal contrast statement: [HF] vs. [HF-MB, HF-HB]. ^5^Orthogonal contrast statement: [HF-MB] vs. [HF-HB]. *ADFI* average daily feed intake, *ADG* average daily gain, *BW* body weight, *SEM* Standard error of mean, *TFI* Total feed intake, *wt* weight

^a, b^ Different letters represent statistically different by the effect of HF

### BCAAs increased fat content and altered relative fatty acid proportions in colostrum and milk

Compared to CON, high fat diets increased the percentage of fat in colostrum (*P* = 0.01) and milk at d 12 and d 18 (*P* < 0.05, Table 4). Similarly, high fat diets increased the percentage of IgG and IgM in d 12 and d 18 milk (*P* < 0.06). Supplementation of BCAAs (HF-MB and HF-HB) increased fat content by 35.8% and 27.3%, respectively, in colostrum compared to HF (*P* < 0.05, Table 4) and did not significantly alter the fat content in d 12 and d 18 milk. Neither fat inclusion nor BCAAs supplementation affected the contents of protein, lactose, and non-fat solids in colostrum and milk. The BCAAs dose did not affect colostrum or milk fat, protein, lactose, non-fat solids or immunoglobulin percentage.

**Table 4:** Effects of branched chain amino acids supplementation on colostrum composition of lactating sows fed high-fat diets^a^

Items^b^	CON	HF	HF-MB	HF-HB	Pooled SEM	*P* value		
						HF effect^c^	BCAAs effect^d^	BCAAs dose effect^e^
Colostrum								
Fat, %	3.86	4.58	6.22	5.83	0.31	0.001	0.008	0.15
Protein, %	18.45	17.38	16.47	17.78	1.04	0.62	0.53	0.47
Lactose, %	2.33	2.26	2.30	2.22	0.15	0.85	0.94	0.46
Non-fat solids, %	20.46	20.45	20.07	20.58	1.07	0.43	0.92	0.85
IgG, g/L	71.88	57.08	65.84	66.92	5.67	0.27	0.36	0.25
IgM, g/L	3.25	3.08	3.46	2.98	0.19	0.23	0.42	0.68
Milk-12 d								
Fat, %	5.21	5.95	6.20	6.29	0.19	0.03	0.35	0.97
Protein, %	5.23	5.48	5.16	5.09	0.34	0.56	0.47	0.92
Lactose, %	5.41	5.72	5.68	5.34	0.13	0.52	0.67	0.63
Non-fat solids, %	10.39	10.21	10.08	10.19	0.06	0.47	0.29	0.38
IgG, g/L	1.34	1.82	1.67	1.72	0.07	0.05	0.21	0.34
IgM, g/L	1.59	2.06	2.31	1.97	0.11	0.03	0.51	0.67
Milk-18 d								
Fat, %	5.90	6.15	6.28	6.34	0.21	0.02	0.42	0.67
Protein, %	5.15	5.27	5.34	5.30	0.36	0.73	0.85	0.74
Lactose, %	5.32	5.66	5.41	5.53	0.06	0.69	0.67	0.71
Non-fat solids, %	10.33	10.28	10.14	10.29	0.07	0.63	0.34	0.49
IgG, g/L	1.20	1.69	1.57	1.67	0.08	0.03	0.51	0.64
IgM, g/L	1.42	1.89	1.77	1.80	0.15	0.02	0.56	0.47

^a^Experimental diets were a corn-soybean meal based diet (CON), CON + 8% soybean oil (HF), HF + medium dose of branch chain amino acids (BCAAs, 0.11% leucine, 0.06% isoleucine and 0.22% valine; HF-MB), and HF + high dose of BCAAs (0.22% leucine, 0.12% isoleucine and 0.44% valine; HF-HB). Control diet contained no soybean oil (CON).^b^In the same row, values with different small letter superscripts mean significant difference (*P* < 0.05). ^c^Orthogonal contrast statement: [CON] vs. [HF, HF-MB, HF-HB]. ^d^Orthogonal contrast statement: [HF] vs. [HF-MB, HF-HB]. ^e^Orthogonal contrast statement: [HF-MB] vs. [HF-HB]. Colostrum was collected within 2 h of birth of the first piglet. *SEM* Standard error of mean. Milk was sampled on d 12 and d 18 of lactation

**Fig. 1:**
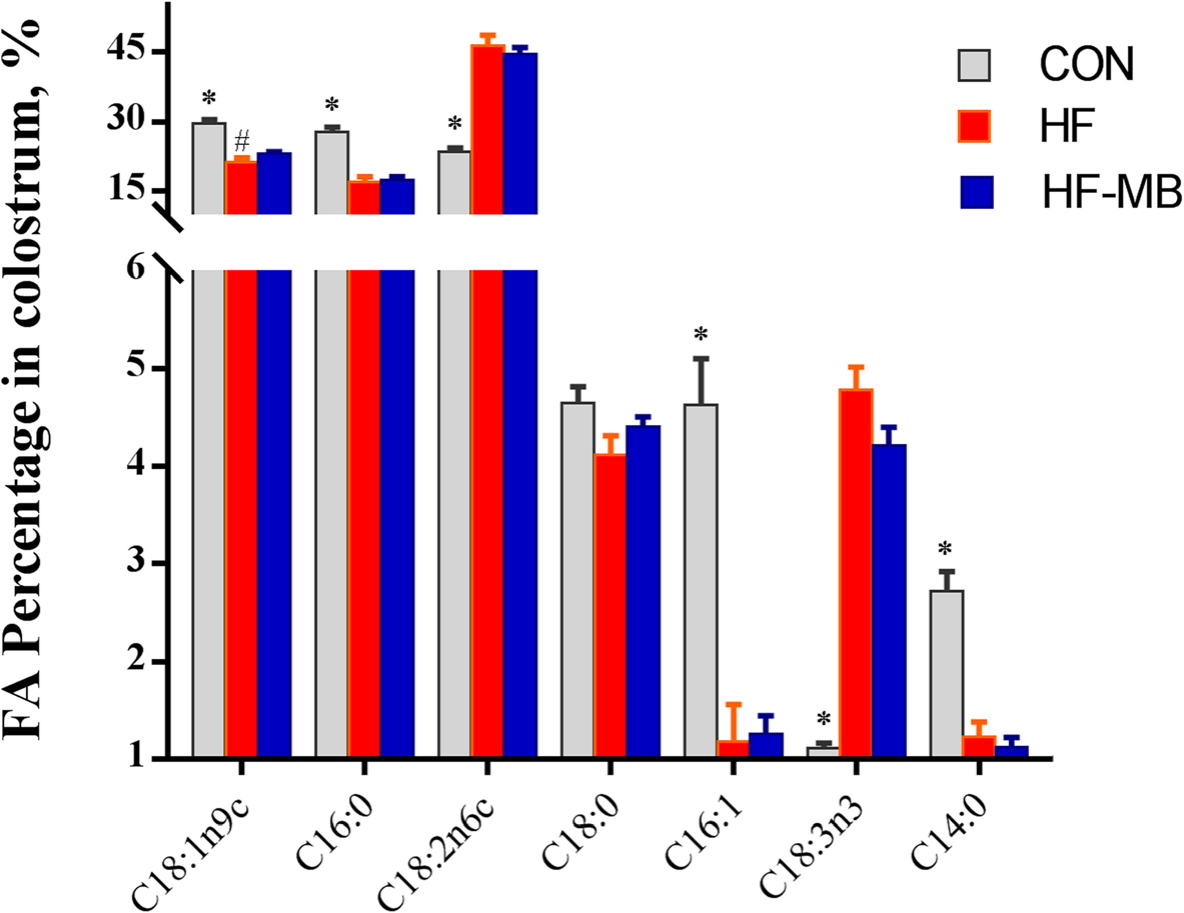
Relative Proportions of the major fatty acids in sow colostrum. Sows were fed with control diet containing no soybean oil (CON), corn-soybean meal based diet containing 8% soybean oil (HF), HF supplemented with medium dose of BCAAs (HF-MB), respectively from late gestation to weaning. BCAAs increased the proportion of C18:1n9c (*P* = 0.030) and tended to decrease the proportion of C18:3n3 (*P* = 0.063). The significantly increased proportion of minor fatty acids included C15:0 (*p* < 0.001), C17:0 (*P* < 0.001), C20:1 (*P* = 0.045) and C20:3n6 (*P* = 0.011). T-test analysis displayed that compared to HF colostrum. HF contained lower percentage of C14:0 (*P* < 0.001) and the sum of C8:0, C10:0 and C12:0 (*P* < 0.01) compared to CON. *denotes difference at *P* < 0.05 between CON and high fat diet groups (HF and HF-MB), and # denotes difference at *P* < 0.05 between HF and HF-MB groups.

The fatty acid profile in colostrum and milk were similar (data not shown) thus only details related to fatty acid profile in colostrum are presented. Similarly, fatty acid profiles from HF-HB sows were similar to that from HF-MB (data not shown). All relevant comparisons were completed with data from HF-MB sows. Relative proportion of the main fatty acids (fatty acid content/total fatty acid content ×100) in colostrum from CON, HF and HF-MB-fed sows is represented in Fig. 1. C18:1n9, C16:0 and C18:2n6 made up more than 80% of total fatty acids (*w*/*w*) in each group. Compared to CON, colostrum from sows fed high fat diets contained a lower proportion of C18:1n9 (21% and 23% vs. 29.6%), C16:0 (17% and 17.4% vs. 27.8%), C16:1 (1.2% and 1.3% vs. 4.6%) and C14:0 (*P* < 0.001). Alternatively, compared to CON, colostrum from sows fed high fat diets had a higher proportion of C18:2n6c (46.3% and 44.4% vs. 23.4%), and C18:3n3 (4.8% and 4.2% vs. 1.1%). Compared to HF, BCAAs in HF-MB increased the proportion of C18:1n9c (*P* = 0.03) and tended to decrease the proportion of C18:3n3 (*P* = 0.06) in colostrum. There was no difference in relative C18:0 proportion among groups.

### Patterns of fatty acid profiles in sow colostrum and serum are differentially altered by BCAAs

Because the colostrum fatty acid profiles between HF-MB and HF-HB were highly similar, we only report the colostrum data from HF-MB for the comparisons of specific fatty acids with HF group. The absolute concentrations (μg/mL) of individual fatty acids in sow colostrum and serum from sows fed HF and HF-MB are shown in Tables 5 and 6, respectively. In colostrum, BCAAs significantly increased the concentration of total fatty acid by 22.1% (*P* = 0.03) compared to the HF group. Concentrations of 6 fatty acids (C15:0, C17:0, C20:3n6, C20:4n6, C20:5n3 and C22:6n3) were increased (*P* = 0.00~0.04) with BCAAs and were odd-chain saturated fatty acids and even-chain n-6 and n-3 PUFA. The concentration of odd-chain fatty acids of C15:0 and C17:0 were increased by 134.8% and 42.8%, respectively, in HF-MB colostrum compared to HF colostrum. Their proportions to the total fatty acids were increased by 90% and 29%, respectively. This indicated an enhanced synthesis of odd-chain fatty acids by BCAAs supplementation.

**Table 5:** Absolute concentrations of Fatty acids in sow colostrum (μg/mL)

Items^a^	HF	HF-MB	Pooled SEM	*P*-value
Higher^b^				
C15:0	39.98	93.89	3.10	0.02
C17:0	71.60	102.22	6.38	0.01
C20:3n6	102.63	136.67	6.23	0.02
C20:4n6	365.08	460.27	25.79	0.04
C20:5n3	39.51	49.31	2.11	0.03
C22:6n3	58.46	86.53	3.41	0.00
Trend of higher^b^				
C20:0	46.87	58.51	3.62	0.08
C21:0	203.86	252.08	12.35	0.07
C22:0	27.70	34.40	1.84	0.05
C16:1	446.40	636.48	65.77	0.07
C18:1n9c	7902.13	10,144.26	692.52	0.08
C20:1	64.45	83.00	6.07	0.10
Unchanged^b^				
C6:0	34.43	25.76	2.84	0.28
C8:0	7.43	1.31	0.43	0.53
C10:0	14.05	13.23	1.78	0.53
C12:0	19.08	20.21	1.58	0.69
C14:0	440.91	542.12	52.18	0.25
C16:0	5979.98	7509.43	552.30	0.11
C18:0	1478.91	1741.14	116.41	0.15
C24:0	179.64	212.16	24.03	0.44
C14:1	7.11	7.83	0.98	0.67
C15:1	14.72	12.58	0.62	0.26
C22:1n9	65.49	63.46	8.71	0.94
C24:1	88.24	107.97	10.05	0.34
C18:2n6c	15,944.54	18,872.16	1192.61	0.20
C18:3n6	124.57	155.77	11.35	0.42
C18:3n3	1613.73	1813.73	127.05	0.40
C20:3n3	52.60	63.84	3.56	0.14
C22:2	38.27	30.72	2.26	0.38
Sum of C20:3n3, C20:5n3 and C22:6n3				
	150.57	199.68	9.14	0.005
Sum of C18:3n6, C20:3n6 and C20:4n6				
	592.28	752.7	39.7	0.041
Total fatty acids				
	35472.37	43331.04	2077.90	0.030

^a^Experimental diets were corn-soybean meal based high diet (HF) and HF supplemented with medium dose of BCAAs (HF-MB). ^b^*T*-test was performed when comparing HF-MB and HF groups. *P* values less than 0.05 was considered statistically different. Colostrum was collected within 2 h from sow mammary gland at parturition. *SEM* Standard error of mean

**Table 6:** Absolute concentrations of fatty acids in sow serum at parturition (μg/mL)

Items^a^	HF^c^	HF-MB^c^	*P*-value
Lower^b^			
C12:0	9.76 ± 1.29	6.13 ± 0.48	0.025
C14:0	14.00 ± 2.18	7.96 ± 0.77	0.026
C15:1	71.55 ± 12.98	27.33 ± 2.26	0.007
C16:0	542.12 ± 74.23	248.82 ± 19.85	0.003
C18:0	444.38 ± 60.89	212.81 ± 13.46	0.004
C18:1n9c	728.28 ± 148.98	335.41 ± 35.41	0.028
C18:2n6c	1071.50 ± 176.86	482.79 ± 69.08	0.011
C20:0	7.45 ± 1.37	4.19 ± 0.30	0.043
C20:1	8.71 ± 1.58	4.19 ± 0.29	0.019
C20:4n6	140.40 ± 22.10	67.30 ± 4.19	0.009
C22:1n9	195.37 ± 6.91	89.43 ± 6.91	0.007
C24:0	31.77 ± 4.77	17.46 ± 1.28	0.016
Trend of lower^b^			
C18:3n3	46.93 ± 5.98	31.47 ± 5.01	0.076
Unchanged^b^			
C16:1	16.64 ± 4.84	10.08 ± 0.68	0.208
C17:0	7.51 ± 0.74	5.00 ± 0.92	0.917
C21:0	8.80 ± 1.29	6.03 ± 1.42	0.179
C22:0	4.39 ± 2.08	4.89 ± 0.52	0.821
C20:3n6	1.70 ± 1.42	1.61 ± 0.83	0.959
C20:5n3	4.63 ± 2.52	4.06 ± 0.93	0.836
C22:6n3	6.92 ± 2.05	4.86 ± 0.41	0.347
C24:1	3.50 ± 1.57	3.41 ± 0.42	0.957
Total Fatty acids			
	3366.30 ± 534.05	1575.23 ± 187.75	0.017

^a^Experimental diets were corn-soybean meal based high fat diet (HF) and HF supplemented with medium dose of BCAAs (HF-MB). ^b^*T*-test was performed when comparing HF-MB and HF groups. ^c^The results were expressed as Mean ± SEM. Because of unequal variances, SEM are reported separately. *P* values less than 0.05 was considered statistically different. Blood was sampled from sow ear vein at farrowing. *SEM* Standard error of mean

Compared to that of HF group, a series of long chain saturated or monounsaturated fatty acids (C20:0, C21:0, C22:0, C16:1, C20:1 and C18:1n9c) showed a tendency to be increased (0.05 < *P* < 0.1) in HF-MB group. C18:3n3 is the precursor of C20:3n3, C20:5n3 and C22:6n3, and C18:3n6, C20:3n6 and C20:4n6 are derived from C18:2n6. Thus, we calculated the total concentrations of these synthesized n-6 or n-3 fatty acids. The results showed that BCAAs enhanced the synthesis of these n-6 and n-3 PUFA by 27.1% and 32.6%, respectively, in colostrum (Table 5). We also tried to detect the odd- and branched- chain fatty acids which are abundant in milk from ruminants including *iso*-14:0, *anteiso*-17:0, *iso*-16:0, *anteiso*-17:0, *iso*-18:0 and *iso*-20:0 [17], but they were below the detection limit of the assay in sow colostrum. All other detectable fatty acids were not different between HF and HF-MB groups (Table 5).

In contrast, in serum, BCAAs in HF-MB significantly decreased the concentration of total fatty acid by 53.2% (*P* = 0.017) compared to the HF group (Table 6). The individual fatty acids that were significantly decreased in HF-MB group included C12:0, C14:0, C16:0, C18:0, C20:0, C24:0, C18:1n9c, and C18:2n6, and consisted mainly of even-chain fatty acids. The concentration of C18:3n3 tended to be lower (*P* = 0.08) in HF-MB group compared to HF; all other detected fatty acids were not different between groups.

**Fig. 2:**
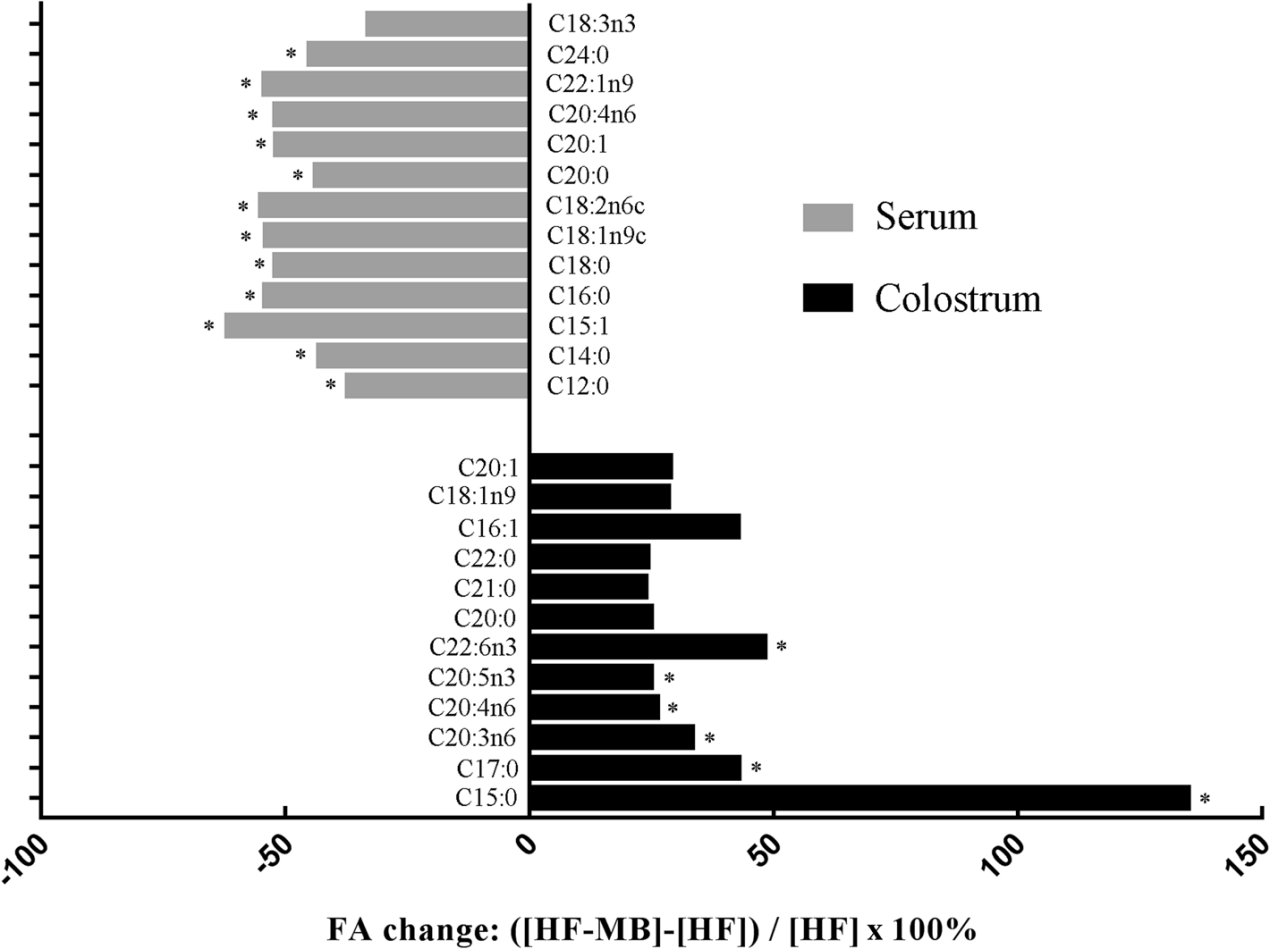
Relative change in fatty acid concentrations in colostrum (grey bars) and sow serum at parturition (black bars) due to BCAAs supplementation. Sows were fed with corn-soybean meal based high fat diet (HF) or HF supplemented with medium dose of BCAAs (HF-MB) from late gestation to weaning. Each bar represents the relative change in fatty acid concentration: ([HF-MB]-[HF])/[HF]×100%. Bars with * represent significant difference between [HF] and [HF-MB], all other bars represent a tendency to be different

To more clearly demonstrate the differentially altered fatty acid profile in colostrum and serum due to BCAAs, the change in colostrum and serum fatty acid profile of HF-MB-fed sows relative to HF-fed sows was calculated (Fig. 2). The patterns of fatty acid profiles in sow colostrum and serum are differentially altered by BCAAs.

## Discussion

Inclusion of fat in sow diets can increase energy intake and decrease body weight loss after weaning. Fat added to sow lactation diets can also increase milk fat content and improve offspring survival rate [2]. To our knowledge, this study was the first to investigate the effects of BCAAs within high fat diets on sow growth and lactation performance under the high fat diet. BCAAs significantly enhanced colostrum fat content and increased piglet body weight and survival rate at weaning. BCAAs and their metabolites have been reported to increase reproductive performance. For example, supplementation of valine and isoleucine in different ratios increased litter weight and milk fat content of mature milk (d 17 of lactation) [12]. In that study, when isoleucine:valine ratio increased milk fat was elevated 15-19%. Supplementation of HMB, the metabolite of leucine, increased milk fat by 40% compared to the unsupplementation group [15]. In both studies, BCAAs or their metabolites supplementation increased litter weights, as well as increased sow body weight loss. In this study, high fat supplementation increased litter weight and survival rate, which was consistent with previous reported. BCAAs with high fat further increased colostrum fat by 30% and increased litter weight by 6.8%. More interestingly, BCAAs reduced sow back fat loss after weaning. *In vitro* study by supplementation of BCAAs to the culture medium of mammary tissue found an elevated level of glutamine and aspartate, which are crucial for the growth, development, and function of the neonatal small intestines [17]. Collectively, the improvement of BCAAs in high fat diets on litter growth, colostrum fat and sow backfat loss are an integrated complex effects of BCAAs, BCAAs metabolites and the fat.

By analysis of the fatty acid composition, we found that dietary fat inhibited *de novo* lipogenesis of the mammary gland (lower percentage of C14:0 and the sum of C8:0, C10:0 and C12:0) which is consistent with published studies in humans and rats (reviewed by [18]). BCAAs significantly increased the absolute concentration of total fatty acids in colostrum (μg/mL). BCAAs also changed the proportions of major fatty acids (C18:3n3 and C18:1n9c), with increased proportions of minor fatty acids C15:0, C17:0, C21:0 and C20:3n6. However, the altered fatty acid profile in sow serum in BCAAs supplementation did not match that in colostrum. In serum BCAAs mainly decreased the absolute concentrations of even-chain saturated fatty acids (C12:0 to C24:0), while in colostrum, sows fed diets with BCAAs increased the absolute concentrations of, if not all, odd-chain fatty acids (C15:0, C17:0) and the long chain PUFA (C20:3n6, C20:4n6, C20:5n3 and C22:6n3). These results indicated that BCAAs may change FA synthesis or supply precursors for the synthesis of the odd chain and minor fatty acids in sow mammary gland during colostrum synthesis.

It is also possible that BCAAs changed the pattern of FA uptake from blood. Milk fat synthesis depends on the availability of fatty acids in the mammary gland. Mammary fatty acids are derived from *de novo* biosynthesis, as well as active uptake of circulating fatty acids originally from diet or released from tissue. Compared to the species of cow, goat and rat, two classic studies revealed that sow mammary gland has much lower extraction rate of blood triglycerides based on calculation from the mammary arteriovenous differences [19, 20], suggesting that the mammary gland of sow synthesize quantitatively more fatty acids than the cow, goat and rat.

The main odd- and branched-chain fatty acids in cow milk are isomers of pentadecanoic acid (C15:0, *iso* C15:0, and *anteiso* C15:0), and heptadecanoic acid (C17:0, *iso* C17:0, and *anteiso* C17:0) [21]. These isomers can also be detected in cattle subcutaneous adipose tissue [22]. Ruminant milk contains much more odd-chain fatty acids than milk from monogastric animals. A very small amount of these isomers is derived from diets because plants only have trace amounts of C15:0 and C17:0 [23]. Ruminant bacteria produce most of the odd-chain fatty acids, in cow milk [24]). Goat mammary gland can also synthesize odd-chain fatty acids, C15:0 and C17:0 [25]. Few studies have reported the presence of odd-chain fatty acids in sow milk. In the present study, we found that in sow colostrum, C15:0 and C17:0 take about 0.30% of all fatty acids, and BCAAs improved their proportion to 0.37%. Different from ruminants, these precursor VFA are not present in sufficient quantity to produce milk fatty acids. One of the possible *de novo* synthesis pathways of odd-chain fatty acids can be achieved by repeated condensation of malonyl-coenzyme A (CoA) with propionyl-CoA as primer to yield linear odd-chain fatty acids [26]. Propionyl-CoA can also be produced in the catabolism of isoleucine and valine. In vitro studies with adipocytes of 3T3L1 revealed that BCAAs contribute to synthesis of odd-chain fatty acids [27]. BCAAs can act as the substrates to produce a series of branched-chain fatty acids by ruminant bacteria (reviewed by [28]), however, the odd- and branched- chain fatty acids, including *iso*-C14:0, *anteiso*-C15:0, *iso*-C16:0, *anteiso*-C17:0, *iso*-C18:0 and *iso*-C20:0, were not detectable in colostrum of sow, a non-ruminant animal, in this study. It indicated a much minor effect of microbes on production of branched-chain fatty acids in sow mammary gland. However, we do not know the contribution of VFA from the hind gut. In the hind gut of sows, fermentation is important under certain circumstances (reviewed by [29]). Thus it cannot be excluded that a shift towards propionate production, under a different balance of gut bacteria, due to fat addition and/or BCAAs could contribute to these variations in odd number fatty acids.

Mammary epithelial cells take up the long chain fatty acids from albumin-bound fatty acids and lipoproteins. The enzyme lipoprotein lipase located in the capillary lumen of the mammary gland hydrolyzes the circulating triglycerides mainly found in VLDL into free fatty acids, which are then taken up by the mammary epithelial cells through transporters [30]. In this study, colostrum was found to possess approximately 10-fold higher concentrations of total fatty acid than the blood, and BCAAs increased this difference to approximately 30-fold. Individual fatty acid comparison showed varying degrees of differences between colostrum and blood. For example, the absolute concentration of α-linolenic acid (ALA, C18:3n3) in serum of HF sows was 46.93 μg/mL, whereas its concentration in the colostrum was 1613.73 μg/mL, a 34-fold difference. However, the concentration of ALA in colostrum was 58-fold higher than in serum of HF-MB group. In addition, a series of fatty acids detected in the colostrum could not be detected in the blood. These fatty acids represented more than 0.8% of total fatty acid in colostrum (*w*/*w*), which included saturated fatty acids (C6:0, C8:0, C10:0, C15:0) and C14:1, which were most likely *de novo* synthesized, and PUFA (C18:3n6, C20:3n3) which were likely converted from dietary ALA (C18:3n3) and linoleic acid (LA, C18:2n6). These observations provide the evidence that the fatty acid compositions in sow colostrum are distinctly different from the FA profile in blood, indicating that the sow mammary gland can selectively synthesize and take up individual FAs at different rates, independent of the blood supply.

BCAAs were reported to reduce hyperlipidemia, hepatic lipid accumulation [8, 31, 32], and obesity (reviewed by [33]) but the intrinsic mechanism was not clear yet. Indirect evidences include in vivo studies that revealed BCAAs upregulate the lipid catabolic genes carnitine palmitoyltransferase I and PPARα [34] and downregulate the hepatic lipogenic genes acetyl-CoA carboxylase α and stearoyl-coAdesaturase 1 in chicks [35]. *In vitro* studies reported BCAAs and its metabolites promote fatty acid oxidation and mitochondrial mass in adipocytes 3T3L1 [36, 37] and enhance fatty acid transport in skeletal muscle stem cells [7]. To our knowledge, there are no reports on BCAAs effect on lipid metabolism in sows. The mechanism is possibly involving the fatty acid catabolism in the mammary gland or other tissues.

The mammary gland is a unique tissue that undergoes repeated cycles of development during and after puberty. The extensive mammary development in pigs occurs during the last third of pregnancy when sow mammary parenchyma tissue (composed of epithelial cells) increases its mass by 200% while the parenchymal lipids decrease by 70% [38]. Studies have revealed that adipocytes in the mammary gland have large plasticity which can reversibly differentiate from adipocytes into secretory epithelia [39] and dedifferentiate from mammary adipocytes to preadipocyte-like precursors during lactation [40]. BCAAs utilization in differentiated 3 T3-L1 adipocytes is dramatically higher than in pre-adipocytes [37]. Furthermore, differentiated adipocytes increased BCAAs catabolic flux for acetyl-CoA pools [41]. Thus, based on the fact that BCAAs act on adipocytes which play actively roles in mammary development, we propose that BCAAs are involved in the mammary structure changes possibly leading to the increase in mammary epithelial cells.

## Conclusions

**Fig. 3:**
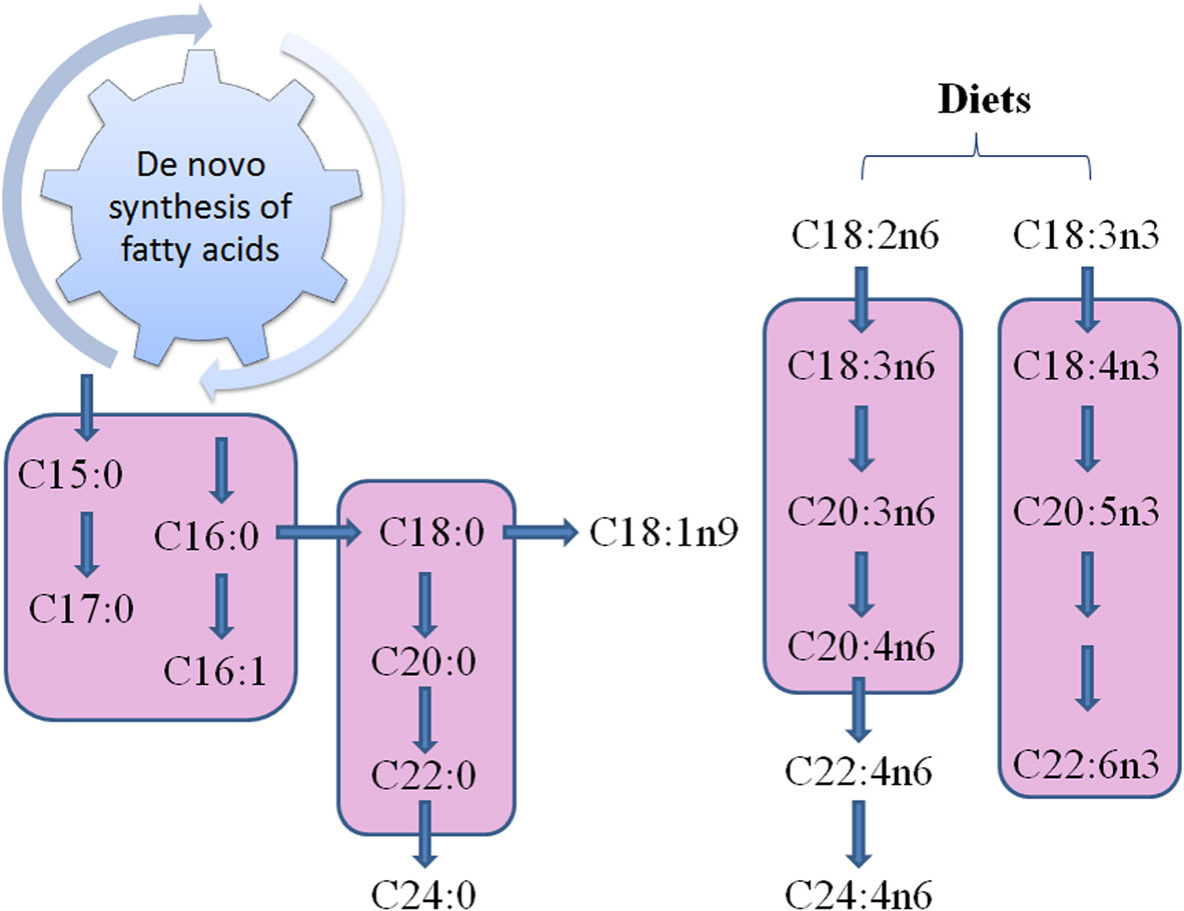
Diagram of milk fatty acid synthesis pathway. The routes with pink background were enhanced by BCAAs in high fat diets

Our present study showed that high fat diet increased litter weights at birth and the survival rate at weaning. BCAAs supplementation in high fat diet increased sow colostrum fat content and body weight of weaned piglets. BCAAs tended to decrease the proportion of major fatty acid C18:3n3 and increased C18:1n9c in colostrum. BCAAs increased the proportions of minor fatty acids C15:0, C17:0, C20:1 and C20:3n6 in colostrum. In addition, sows fed diets with BCAAs decreased the concentration of total fatty acid in serum but increased total fatty acids in colostrum. The comparisons of fatty acid profiles between colostrum and sow serum with or without BCAAs revealed active synthesis or uptake of fatty acids in sow lactation mammary gland (Fig. 3) and dietary BCAAs have profound effect on these processes.

## Supplementary information

**Additional file 1: Figure S1.** The concentrations of total fatty acids in mammary glands of female rats fed with BCAAs.

## Data Availability

The data analyzed during the current study are available from the corresponding author on reasonable request.

## Abbreviations

ADFI: Average daily feed intake
ADG: Average daily weight gain
ALA: α-Linolenic acid
BCAAs: Branched chain amino acids
BCKA: Branched-chain α-keto acids
BCKD: BCKA dehydrogenase complex
CP: Crude protein
HF: High fat
LA: Linoleic acid
MUFA: Monounsaturated fatty acids
PPARα: Peroxisome proliferator-activated receptor α
PUFA: Poly unsaturated fatty acids
SFA: Saturated fatty acids
