# Supplementary material for: Branched chain amino acids alter fatty acid profile in colostrum of sows fed a high fat diet
Source: J Anim Sci Biotechnol. 2020 Feb 17;11:9. doi: 10.1186/s40104-019-0423-9 (PMC7025410; doi:10.1186/s40104-019-0423-9)

Supplemental Materials

**Legend**

Figure S1

The concentrations of total fatty acids in mammary glands. Female rats were fed with high fat (HF) with varying doses of BCAAs (HF-LB, HF-MB and HF-HB) during gestation and lactation. The treatment with no BCAAs supplement in AIN-93G was set as the negative control (CON). BCAAs supplemented with drinking water (HF-LB: low dose of BCAAs; HF-MB: medium dose of BCAAs; HF-HB: high dose of BCAAs). Medium dose of BCAAs was exactly set as our previous published study [1]. Low dose was set as half of the medium dose, and high dose was set as double of the medium dose. Data were analyzed using one-way analysis of variance in accordance with the general linear model procedures of SAS 9.4 (SAS Institute 182 Inc., Cary, NC, USA). Statistical differences among mean values were assessed using Duncan’s multiple range test. Different letters represent significant statistical differences among treatments. Significance level was set at *P* < 0.05.

Reference:

1. Appl. Physiol. Nutr. Metab. 38: 836–843 (2013) dx.doi.org/10.1139/apnm-2012-0496

Figure S1:


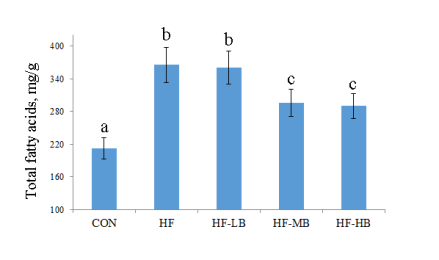

Supplement: Supplementary file 1 — Additional file 1: Figure S1. The concentrations of total fatty acids in mammary glands of female rats fed with BCAAs. [file 40104_2019_423_MOESM1_ESM.docx]
